# Conservation genetics and potential geographic distribution modeling of *Corybas taliensis*, a small ‘sky Island’ orchid species in China

**DOI:** 10.1186/s12870-023-04693-y

**Published:** 2024-01-02

**Authors:** Yuhang Liu, Huichun Wang, Jing Yang, Zhiling Dao, Weibang Sun

**Affiliations:** 1 Yunnan Key Laboratory for Integrative Conservation of Plant Species with Extremely Small Populations, Kunming Institute of Botany, Chinese Academy of Sciences (CAS), Kunming, Yunnan 650201 China; 2 Key Laboratory for Plant Diversity and Biogeography of East Asia, Kunming Institute of Botany, Chinese Academy of Sciences (CAS), Kunming, Yunnan 650201 China; 3 University of Chinese Academy of Sciences, Beijing, 100049 China; 4 Kunming Botanical Garden, Kunming Institute of Botany, Chinese Academy of Sciences (CAS), Kunming, Yunnan 650201 China

**Keywords:** *Corybas taliensis*, ddRAD-seq, Population genetic, MaxEnt, Suitable habitat, Conservation management

## Abstract

**Background:**

*Corybas taliensis* is an endemic species of sky islands in China. Its habitat is fragile and unstable, and it is likely that the species is threatened. However, it is difficult to determine the conservation priority or unit without knowing the genetic background and the overall distribution of this species. In this study, we used double digest restriction-site associated DNA-sequencing (ddRAD-seq) to investigate the conservation genomics of *C. taliensis*. At the same time, we modeled the extent of suitable habitat for *C. taliensis* in present and future (2030 and 2090) habitat using the maximum-entropy (MaxEnt) model.

**Results:**

The results suggested that the related *C. fanjingshanensis* belongs to *C. taliensis* and should not be considered a separate species. All the sampling locations were divided into three genetic groups: the Sichuan & Guizhou population (SG population), the Hengduan Mountains population (HD population) and Himalayan population (HM population), and we found that there was complex gene flow between the sampling locations of HD population. MT was distinct genetically from the other sampling locations due to the unique environment in Motuo. The genetic diversity (π, *H*_*e*_) of *C. taliensis* was relatively high, but its contemporary effective population size (*N*_*e*_) was small. *C. taliensis* might be currently affected by inbreeding depression, although its large population density may be able to reduce the effect of this. The predicted areas of suitable habitat currently found in higher mountains will not change significantly in the future, and these suitable habitats are predicted to spread to other higher mountains under future climate change. However, suitable habitat in relatively low altitude areas may disappear in the future. This suggests that *C. taliensis* will be caught in a ‘summit trap’ in low altitude areas, however, in contrast, the high altitude of the Himalaya and the Hengduan Mountains are predicted to act as ‘biological refuges’ for *C. taliensis* in the future.

**Conclusions:**

These results not only provide a new understanding of the genetic background and potential resource distribution of *C. taliensis*, but also lay the foundation for its conservation and management.

**Supplementary Information:**

The online version contains supplementary material available at 10.1186/s12870-023-04693-y.

## Background

Habitat deterioration and destruction caused by anthropogenic stresses and global climate change threaten global ecological function [1–3], and result in the continuing decline of the world ‘s biodiversity [4, 5]. A thorough assessment of the risks facing biodiversity is needed to plan action to slow rates of species and biodiversity decline, and to protect and manage biological resources [6]. Endangered species are more likely to become extinct under global climate change and anthropogenic disturbance, and therefore require more attention and research in a timely manner to prevent their loss [7, 8]. In addition to reproductive difficulties, small populations of endangered species are also affected by genetic drift, which can erode the genetic diversity of populations and can lead to a rapid increase in genetic differentiation among populations [9–11]. The loss of genetic diversity may reduce the persistence of populations, as well as reducing the evolutionary potential of the species as a whole, and high genetic differentiation may lead to the decline of outbreeding depression [12–14]. The ecological resilience (the ability to resist or recover from disturbance, also called ecological robustness) and the ability of organisms to adapt to environmental changes are also affected by the underlying genetic characteristics of their populations [15]. Therefore, conservation genetics research is also important for the development of conservation priorities and management strategies for endangered species. Meanwhile, understanding the geographical distribution of threatened taxa is a crucial step towards their conservation [16], and information regarding the potential geographical distributions of species based on biological-climate relationships can help with decision-making in plant protection projects [17].

The concepts of island biogeography, as originally proposed by MacArthur and Wilson (1967), and their later development gave rise to the idea of “sky islands” to describe continental habitat (e.g., mountains) that are isolated from similar habitat by unfavorable terrain (e.g., valleys) [18], in the same way that the sea separates oceanic islands [19]. Species in sky islands face unsuitable climate in the intervening valleys, and have restricted movement from one montane area to another [20]. This means that the sky islands are not well connected and that there is little migration of individuals through the habitat barrier [21], preventing gene flow between populations in different sky islands, and eventually meaning that these populations have high levels of inter-population genetic divergence and unique patterns of genetic structure [19, 22]. However, the gene flow restriction among sky island species is not absolute, and is related to the actual spatial distribution, natural conditions and the mobility of the species themselves. Some sky island species have reduced gene flow among populations but are not completely isolated from one another, as is the case of the New Mexico ridge-nosed rattlesnake, *Crotalus willardi obscurus* [23], the black bear *Ursus americanus* [24], the alpine plant *Sedum lanceolatum* [25] and the montane grasshopper *Melanoplus oregonensis* [26]. Sometimes sky island species show low genetic differentiation between populations, which may be because they share the same recent origin and have not yet had time to develop pronounced structural differentiation between populations. For example, the nine island populations of gazelles (Bovidae: Antilopinae) studied by Chiozzi et al., showed low genetic differentiation from their mainland relatives after gene flow caused by spontaneous colonization had been excluded, indicating that they were of recent origin [27]. Because species responses to climatic changes are influenced by interacting factors such as ecology, landscape topography, and latitude and longitude, the pattern and tempo of diversification will vary [28]. For example, the presence of a species on multiple sky islands could result from the fragmentation of a wide-ranging common ancestor or may be due to inter-island dispersal [ 29]. Within the sky islands themselves, changing climatic conditions can cause suitable environments for species or populations to shift, expand or contract along elevational gradients [29, 30]. This allows these islands to serve as refugia and/or to generate strong ecological gradients [31], and that in turn may allow long-term population persistence during global climate fluctuations in the mountains [32]. Montane habitat therefore often become hotspots of endemism, intraspecific genetic diversity and species richness, and provide opportunities to study how different evolutionary processes lead to diversification [33]. Sky island systems are thus attractive study areas for investigating how heterogeneous landscapes promote population genetic differentiation and speciation, both spatially and temporally [34, 35].

**Fig. 1:**
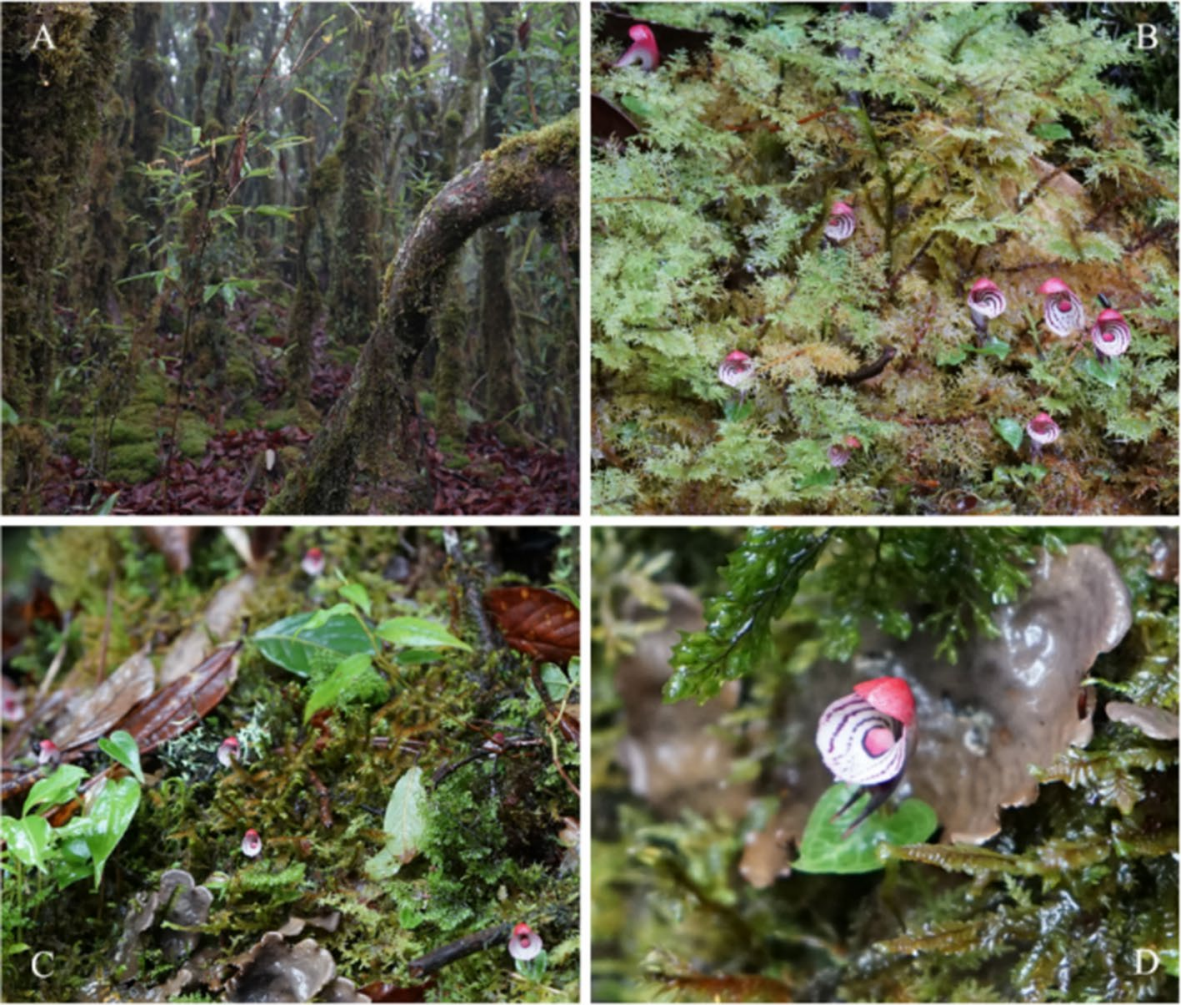
*Corybas taliensis* in the wild. The habitat (**A**), habitat and flowers (**B**, **C**), and flower (**D**)

*Corybas Salisb.* is a genus in the Orchidaceae, originally published by the British botanist Richard Anthony Salisbury in 1807 [36]. About 160 species are known globally, and are mainly distributed from the Himalayas in the north to Macquarie Island, which is about half way between Australia and Antarctica (GBIF, https://www.gbif.org/). Corybas has centers of diversity in New Guinea, Australia and New Zealand, and China is the northern margin of the genus [37]. *C. taliensis* was first published in 1951 by Tang and Wang in China. The type specimens were collected from Dali, Yunnan, and are now deposited in the Museum of Biological Specimens, Peking University, Beijing (PEY; PEY0051469) [38]. Through field investigations, we found nine wild locations of *C. taliensis* in Xizang, Yunnan, Sichuan and Guizhou, which were narrowly restricted to isolated montane regions, mainly between elevations of 2300 and 3500 m. *C. taliensis* can therefore be considered to be a sky island species [39]. Most plants were found in clumps of moss under coppiced montane moss forests, or coniferous and broad-leaved mixed forests, with a few plants found growing in the deciduous layer with thick humus or wet rotten wood (Fig. 1). The relative fragility of such habitat, coupled with their location on isolated and complex sky islands, makes the species more vulnerable to stochastic effects and local extinctions [40]. *C. taliensis* has been categorized as Endangered (EN) in both the Threatened Species List of China’s Higher Plants [41] and the Chinese Red List of biodiversity-vascular plant volume published in 2020 [42], and has been listed as second-class protected plant of the state in the National key protected wild plants list of China in 2021 [43].

The *C. taliensis* localities which have been investigated in the field and constitute our sampling locations include: Dingjie, Zhari, Motuo, Pianma, Tengchong, Eryuan, Cangshan, Puge, abbreviated as: DJ, ZR, MT, PM, TC, EY, CS, PG, respectively. It is worth noting that *C. fanjingshanensis* was published in 2007 as a new species of *Corybas* from Guizhou [37]. However, further research and investigation speculated that this species may in fact belong to *C. taliensis* rather than representing a new species [44]. In this study, we named the *C. fanjingshanensis* sampling location GZ and analyzed it together with the identified *C. taliensis* from other sampling locations.

In this study, double digest restriction-site associated DNA-sequencing (ddRAD-seq) was selected to conduct conservation genetics research on *C. taliensis.* ddRAD-seq is widely used in plant population genetics, phylogenetics, phylogeography and molecular breeding [45, 46] as a simple, fast and inexpensive method of constructing RAD-Seq databases [47]. We used MaxEnt [48] to predict the current and future distribution of habitat that are potentially suitable for *C. taliensis*. MaxEnt can deal with presence-only data and has been shown to outperform most other models, even the ensemble model [49, 50], and performs better than other species distribution models when analyzing data with small sample sizes [51, 52].

The objectives of this study are (1) to explore the population structure in *C. taliensis*, to infer the levels of gene flow among populations, and to estimate the genetic diversity and contemporary effective population size (*N*_*e*_) of each population; (2) to analyze the possible reasons underlying the observed genetic patterns in *C. taliensis* as a sky island species; and (3), to use the MaxEnt to predict areas of potentially suitable habitat for *C. taliensis* under current and future climate scenarios. We then aim to analyze the population genetic characteristics of *C. taliensis*, predict the future changes of its suitable habitat, and put forward suggestions for protection and management.

## Results

### Processing of sequencing data

A total of 542 million reads were generated from all samples across the 9 sampling locations (DJ, ZR, MT, PM, TC, EY, CS, PG, GZ), and after filtering out low-quality reads and reads without RAD-tags, 528 million reads remained for processing. The sequencing depth of samples ranged from 5.90× (CS05) to 28.80× (GZ14), with an average coverage of 13.32× (Table S1). We used Stacks to carry out de novo analysis on *C. taliensis* [53], through SNP calling with the values of the parameters M, m and n, set to M = 1, m = 2, n = 2, which resulted in 5116 single nucleotide polymorphisms (SNPs; Tables S2, S3). Tajima’s *D* statistical test showed a negative average of Tajima’s *D* values (-0.369) with a high degree of significance (p-value < 0.05) for 5116 SNPs, of which 4222 were significantly negative (Fig. S1).

### Population structure and genetic diversity

**Fig. 2:**
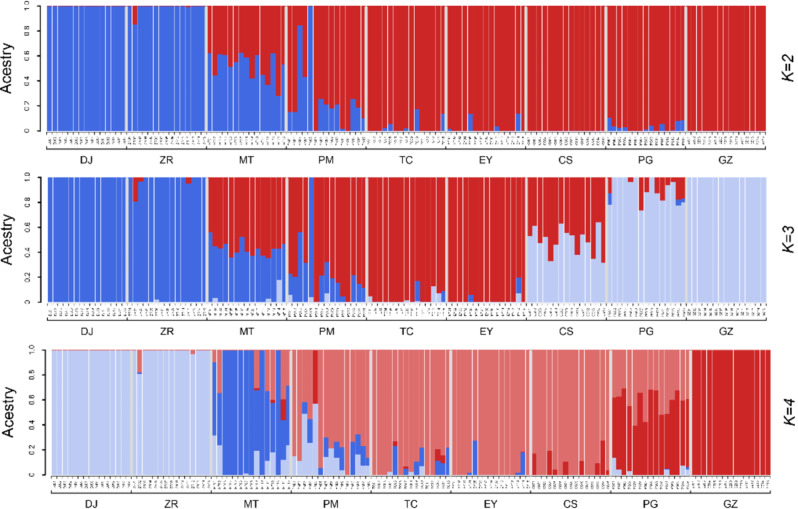
Bayesian cluster analysis result (*K* = 2, *K* = 3, *K* = 4). When *K* = 4, ZR and DJ clustered together and were together defined as the HM population, PG and GZ clustered together and were defined as the SG population. MT was suggested to be an independently segregated group. All the other sampling locations (CS, EY, TC, MT and PM) made up the YN population. When *K* = 2, all sampling locations were divided into two groups: ZR and DJ clustered together, and the others clustered together. When *K* = 3, all sampling locations were divided into three groups, including group1 (DJ, ZR), group2 (MT, PM, TC, EY) and group3 (CS, PG, GZ).

**Fig. 3:**
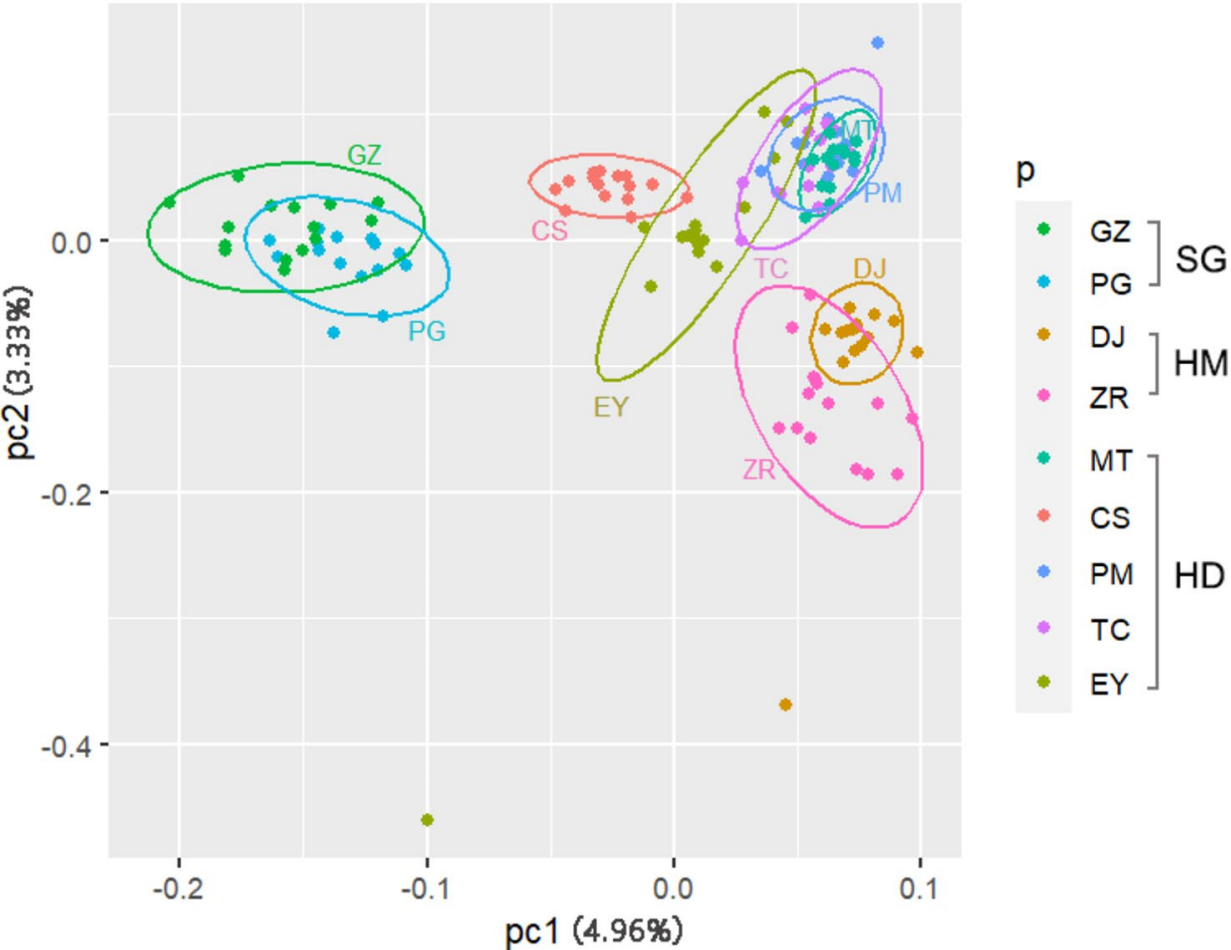
The PCA analysis of genetic variation among different *Corybas taliensis* poopulations. Sampling locations: DJ(Dingjie), ZR(Zhari), MT(Motuo), PM(Pianma), TC(Tengchong), EY(Eryuan), CS(Cangshan), PG(Puge), GZ(Guizhou). Populations: SG (Sichuan & Guizhou population), HD (Hengduan Mountains population), HM (Himalaya population). The low values of PC1 and PC2 indicated that the difference between the sampling locations is not significant. Grouping populations by the intersection of 95% confidence ellipses. ZR and DJ clustered together and were defined as the HM population, while PG and GZ clustered together and were defined as the SG population. All the other sampling locations (CS, EY, TC, MT and PM) made up the HD population

From the CV error values, the optimal *K* value was found to be 4 (Fig. S2). Bayesian cluster analysis results at *K* = 4 showed that DJ, ZR and PG, GZ formed two independent genetic groups, while PM, TC, EY and CS formed a genetic group (named the “YN population”, as all samples were from Yunnan Province), and with MT on its own as a separate group (Fig. 2). The population structures resulting from the Bayesian cluster analyses at *K* = 2, *K* = 3 and *K* = 4 show that MT is an intermediate state between DJ, ZR and the other sampling locations. In the principal component analysis (PCA) analysis, the cumulate reliabilities of PC1 and PC2 (the first two principal components) were just 8.29%, indicating that the differences between the sampling locations were not significant. We were therefore only able to group populations by the intersection of the 95% confidence ellipses. All samples could be separated into one of three groups (Fig. 3). ZR and DJ clustered together and were defined as the HM population, while PG and GZ clustered together and were defined as the SG population. All the other sampling locations (CS, EY, TC, MT and PM) made up HD population. The relationship between the HMand HD populations was close, and both fell relatively far away from the SG population in the PCA analysis. MT fell into a separate group in the Bayesian cluster analysis, but was in the same group as CS, EY, TC and PM in the PCA. In both methods, GZ clustered with PG. The individuals in PG were determined as *C. taliensis*, and those from GZ may therefore be the same species as those in PG. BayesAss analysis showed that there was no obvious recent gene flow among sampling locations (Table 1). The results of the Mantel tests showed the correlations between genetic, geographic, environmental, and environmental resistance distances. Among these linear relationships, the geographic and environmental resistance distances were significantly correlated with genetic distance (IBD: R^2^ = 0.3049, p = e^− 4^; IBR_env_: R^2^ = 0.3079, p = 2e^− 4^) (Fig. S3). The fixation index (*F*_*ST*_) between DJ and PG was 0.121, and that between DJ and GZ was 0.114 (Table 2). The results of AMOVA analysis showed that among the total genetic diversity of the populations (SG, HM and HD) analyzed by PCA, 0.67% was attributable to differences among populations, -1.99% was attributable to sampling locations differences, and 101.32% was attributable to intra-population differences. In the total genetic diversity of the populations (SG, HM, MT and YN) from Bayesian clustering analysis, -0.23% was attributable to differences among populations, -1.37% was attributable to sampling locations differences, and 101.60% was attributable to intra-population differences (Table S4).

**Table 1:** Recent migration rates between each pair of sampling locations

sampling location	PG	DJ	ZR	PM	TC	EY	GZ	MT	CS
PG	0.8473 (0.0332)	0.0139 (0.0135)	0.0136 (0.0131)	0.0137 (0.0130)	0.0137 (0.0132)	0.0556 (0.0248)	0.0141 (0.0135)	0.0141 (0.0135)	0.0139 (0.0133)
DJ	0.0140 (0.0131)	0.8468 (0.0329)	0.0140 (0.0132)	0.0136 (0.0130)	0.0139 (0.0133)	0.0561 (0.0247)	0.0137 (0.0131)	0.0137 (0.0131)	0.0141 (0.0136)
ZR	0.0138 (0.0132)	0.0142 (0.0136)	0.8893 (0.0311)	0.0139 (0.0134)	0.0134 (0.0130)	0.0140 (0.0135)	0.0139 (0.0134)	0.0136 (0.0132)	0.0139 (0.0135)
PM	0.0139 (0.0133)	0.0141 (0.0136)	0.0139 (0.0132)	0.8749 (0.0325)	0.0279 (0.0185)	0.0141 (0.0135)	0.0137 (0.0133)	0.0138 (0.0130)	0.0136 (0.0130)
TC	0.0140 (0.0135)	0.0139 (0.0133)	0.0138 (0.0133)	0.0140 (0.0133)	0.8886 (0.0311)	0.0136 (0.0132)	0.0141 (0.0135)	0.0138 (0.0133)	0.0142 (0.0133)
EY	0.0140 (0.0133)	0.0139 (0.0135)	0.0136 (0.0131)	0.0273 (0.0182)	0.1113 (0.0317)	0.7784 (0.0311)	0.0138 (0.0131)	0.0138 (0.0134)	0.0138 (0.0133)
GZ	0.0139 (0.0134)	0.0139 (0.0136)	0.0140 (0.0136)	0.0141 (0.0137)	0.0137 (0.0135)	0.0135 (0.0131)	0.8893 (0.0318)	0.0138 (0.0133)	0.0136 (0.0132)
MT	0.0139 (0.0132)	0.0140 (0.0134)	0.0135 (0.0129)	0.0139 (0.0130)	0.0138 (0.0134)	0.0137 (0.0134)	0.0141 (0.0137)	0.8894 (0.0313)	0.0136 (0.0130)
CS	0.0139 (0.0133)	0.0140 (0.0133)	0.0140 (0.0136)	0.0138 (0.0131)	0.0142 (0.0134)	0.0140 (0.0133)	0.0138 (0.0133)	0.0139 (0.0132)	0.8884 (0.0317)

**Table 2:** **Genetic differentiation coefficient** (***F***_***ST***_) **between each pair of sampling locations**

Sampling location	DJ	ZR	PM	TC	EY	GZ	MT	CS
PG	0.121	0.099	0.112	0.102	0.109	0.084	0.110	0.082
DJ		0.080	0.094	0.085	0.104	0.114	0.090	0.090
ZR			0.078	0.072	0.083	0.094	0.073	0.080
PM				0.056	0.080	0.107	0.076	0.074
TC					0.069	0.098	0.069	0.064
EY						0.104	0.088	0.075
GZ							0.103	0.079
MT								0.078

The number of individuals (N) investigated in the wild was highest in DJ and TC,and there were fewer individuals at PM, MT, ZR, PG and GZ (Table 3). In the population genetic statistics, the nucleotide diversity (π) values were not significantly different among sampling locations, populations, or species. The observed heterozygosity (*H*_*o*_) at different grouping levels ranged from 0.0308 to 0.0553, and the expected heterozygosity (*H*_*e*_) values ranged from 0.0690 to 0.1005. The inbreeding coefficients (*F*_*IS*_) were significantly greater than 0 (p = 4.151e^− 6^ < 0.05) NeEstimator V2.1 was not able to obtain reliable *N*_*e*_ estimates (inf) for ZR, PM, TC, EY, GZ, MT and CS, but *N*_*e*_ estimates for the other four sampling locations are given in Table 3

**Table 3:** Summary of genetic diversity, effective population size and the number of individuals in different populations and sampling locations of *Corybas taliensis*

Genetic groups	Sampling locations	Samples	*H*_*o*_	*H*_*e*_	π	*F*_*IS*_	*N*_*e*_ *(95%C.I.)*	N
**SG**	**-**	**30**	**0.0471**	**0.0915**	**0.0939**	**0.1985**	**4.7 (2.8, 7.7)**	**1260**
	PG	15	0.0467	0.0757	0.0799	0.1047	16.3 (2.5, inf)	560
	GZ	15	0.0397	0.0867	0.0919	0.1745	Inf (15.5, inf)	700
**HM**	**-**	**30**	**0.0355**	**0.0996**	**0.1025**	**0.3222**	**64.8 (16.3, inf)**	**9500**
	DJ	15	0.0495	0.0738	0.0780	0.0918	17.7 (2.7, inf)	9000
	ZR	15	0.0396	0.0972	0.1031	0.2142	Inf (9.9, inf)	500
**HD**	**-**	**75**	**0.0421**	**0.0954**	**0.0964**	**0.4052**	**23.4 (16.9, 33.0)**	**20,414**
	PM	15	0.0308	0.0789	0.0839	0.1712	Inf (31.0, inf)	107
	TC	15	0.0359	0.0825	0.0870	0.1723	Inf (17.1, inf)	9000
	EY	15	0.0341	0.0766	0.0834	0.1435	Inf (1.6, inf)	5000
	MT	15	0.0421	0.0784	0.0830	0.1291	76.4 (10.8, inf)	307
	CS	15	0.0553	0.0690	0.0723	0.0677	14.5 (4.9, 235.4)	6000
**all**	**-**	**135**	**0.0418**	**0.1005**	**0.1011**	**0.5878**	**28.7 (23.5, 34.9)**	**31,174**

### Demographic inference

**Fig. 4:**
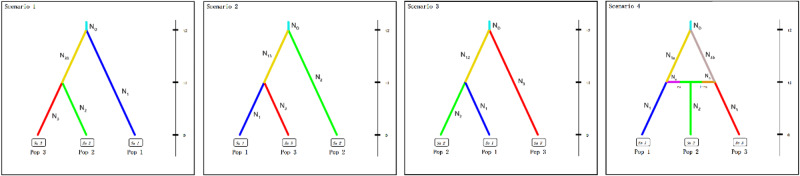
Scenarios tested in DIYABC. t1 and t2 represents the time scale by the generation, and N_#_ represents the effective population size in each genetic group. Pop1, Pop2 and Pop3 correspond to SG population, HD population and HM population, respectively

The DIYABC analysis supports the demographics of scenario 4 (Fig. 4), which is most suitable for the three populations. This scenario was supported by the model (votes = 1091; posterior probability p = 0.757). Other scenarios include scenario1, scenario2 and scenario3, whose votes are 802, 51 and 56. Under scenario 4, The medians of N_1a_, N_3b_, N_1_, N_2_ and N_3_ were 5062 (95% CI: 1147–9348), 3312 (95% CI: 527–8963), 3316 (95% CI: 554–9060), 5102 (95% CI: 1233–9501) and 4923 (95% CI: 1003–9534) respectively, and were higher than N_0_ (1041 with 95% CI of 461–3781) (Table 4). While principal component analysis (PCA) showed that the observed data set belongs to the point cluster of posterior distribution (Fig. S4). Therefore, we think that the selected scenario fits with the observed data.

**Table 4:** Parameter estimation of selected scenario 4 when using random forest prediction in DIYABC, including expectation, median and 90% confidence interval

Parameter	Expectation	Median	Quantile_0.05	Quantile_0.95
N_0_	1431	1041	461	3781
N_1a_	5260	5062	1147	9348
N_3b_	3982	3312	527	8963
N_1_	3896	3166	554	9060
N_2_	5293	5102	1233	9501
N_3_	5214	4923	1003	9534
t1	760	799	399	987
t2	763	799	402	987
ra	0.217116	0.181953	0.0228036	0.568217

### Risk of non-adaptedness

**Fig. 5:**
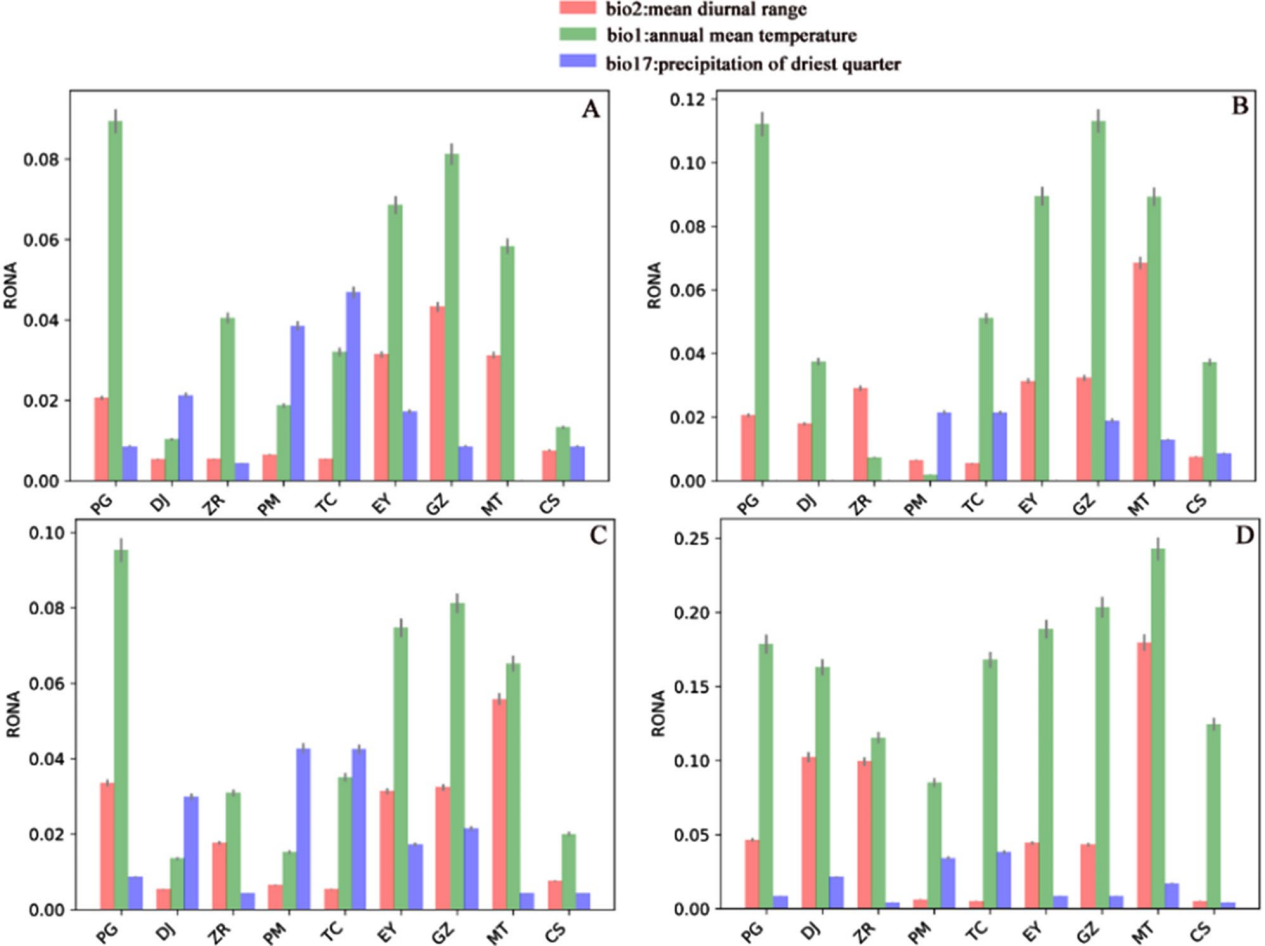
Risk of non-adaptedness plot for the three bioclim factors with most associations under SSP126 and SSP585 scenarios in 2030 and 2090. (**A**) the plot for SSP126 in 2030; (**B**) the plot for SSP126 in 2090; (**C**) the plot for SSP585 in 2030; (**D**) the plot for SSP585 in 2090

The RONA analysis summary for 2030 and 2090 under low (SSP126) and high (SSP585) emission scenarios can be found in Fig. 5 and Table S5. The most representative environmental variables were found to be “bio2-mean diurnal range” (199 SNPs, average R^2 = 0.1395), ”bio1-annual mean temperature” (153 SNPs, average R^2 = 0.1808) and “bio17-precipitation of driest quarter” (150 SNPs, average R^2 = 0.0524). There was no significant difference in the values of RONA at each site for SSP126 and for SSP585 in 2030. In 2090, most RONA values for SSP585 were greater than those for SSP126, and, in most cases, the highest RONA values were for bio1. Under the SSP126 predictions, GZ had the highest RONA value for bio2 (0.043210), PG had the highest for bio1 (0.089439), and TC the highest for bio 17 (0.046819) in 2030. The highest RONA value for bio2 was at MT (0.068510), for bio1 was at GZ (0.113150), and that of bio17 was PM (0.021513) in 2090. Under the SSP585 predictions, MT had the highest RONA value for bio2 (0.055839), PG that for bio1 (0.095337), and PM that for bio 17 (0.042791) in 2030. The highest RONA value in 2090 for bio2 was found at MT (0.79667), for bio1 at MT (0.242849), and for bio17 at TC (0.038208).

### Data preparation for prediction of habitat suitable for *Corybas taliensis*

**Fig. 6:**
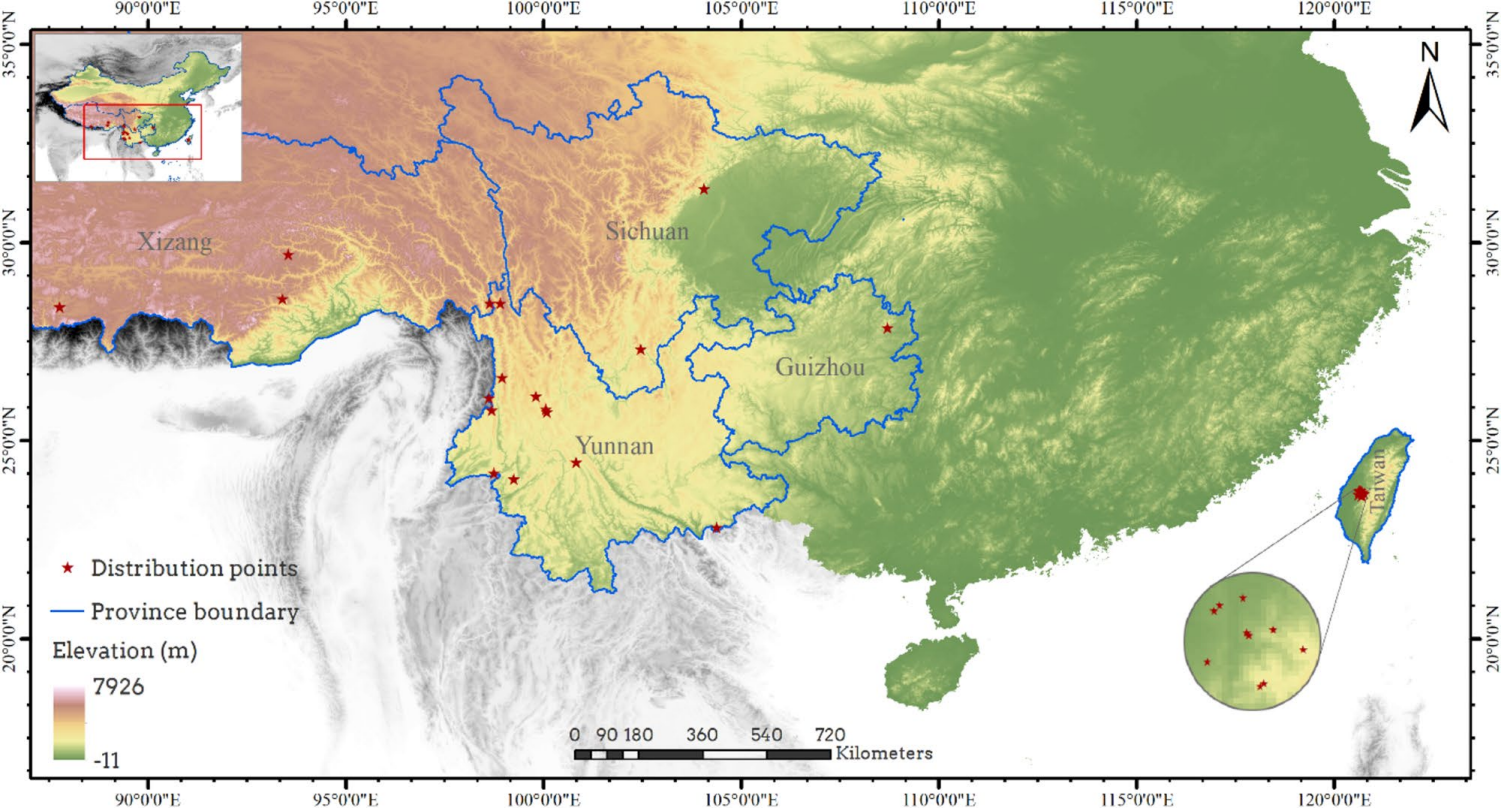
The 28 *Corybas taliensis* distribution points used for modeling potential geographic distribution. *Note*: More information about distribution points can be found in Table S6, Province boundaries were taken from theChina standard map GS(2019)1822 without modification, and Elevation data were downloaded from www.worldclim.org

After processing and screening, we found that 28 distribution points were suitable for use in the prediction of suitability of habitat (Fig. 6). Ten environmental factors were selected for MaxEnt modeling, including four climate factors: (isothermality (bio3), temperature seasonality (bio4), temperature annual range (bio7), precipitation of driest month (bio14)), and six soil factors (sand percentage of topsoil (T_SAND), silt percentage of topsoil (T_SILT), bulk density of topsoil (T_BULK_DEN), organic carbon percentage of topsoil (T_OC), total exchangeable base of topsoil (T_TEB), bulk density of subsoil S_BULK_DEN)).

### Prediction of the potential geographical distribution of *Corybas taliensis*

**Fig. 7:**
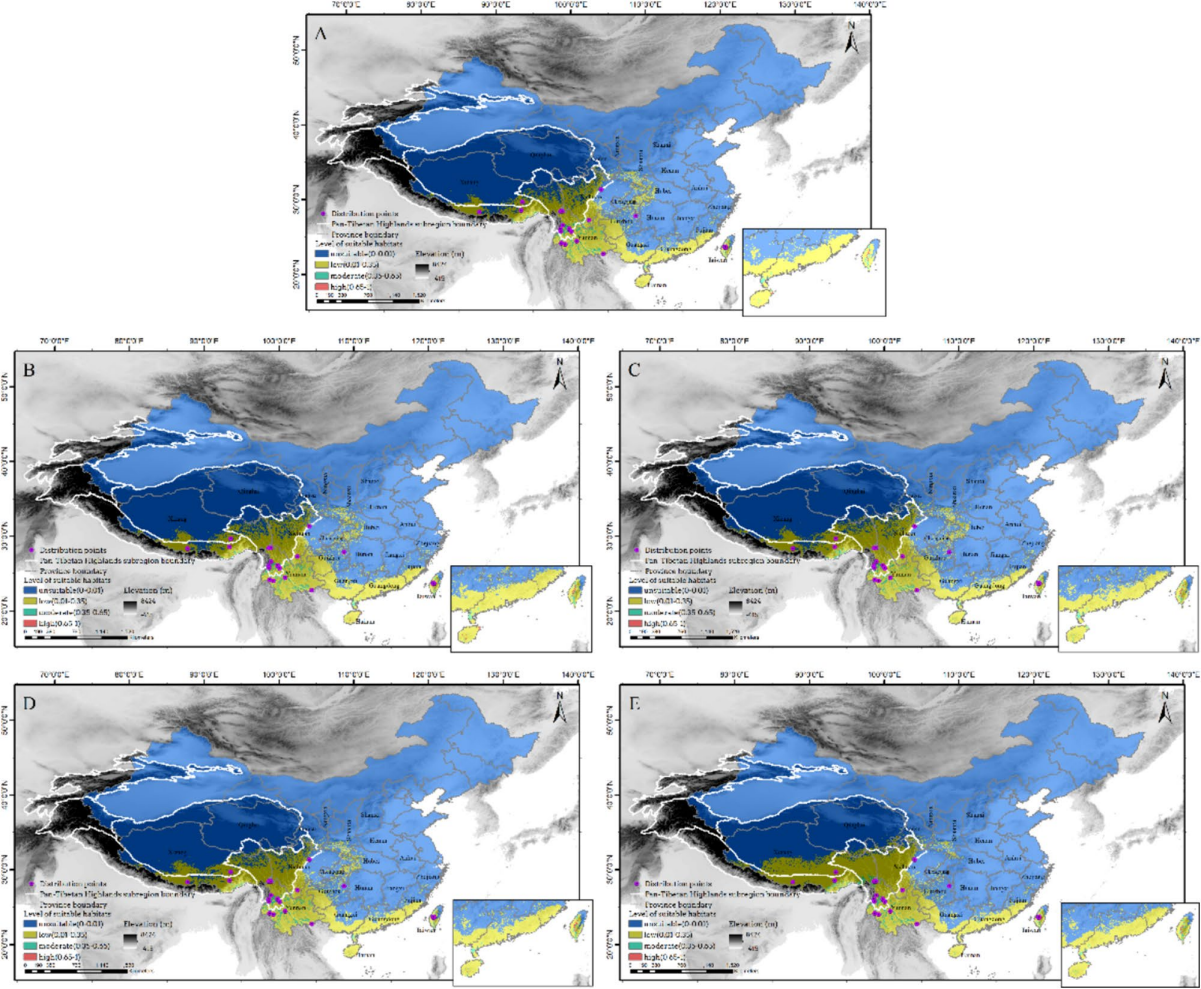
Distribution map of current and future habitat predicted to be suitable for *Corybas taliensis*. Current distribution of suitable habitat (**A**), habitatDistribution of suitable habitat in 2030 (**B**) and 2090 (**C**) under the SSP126 climate scenario, and in 2030 (**D**) and 2090 (**E**) under the SSP585 climate scenario. *Note*: More information about the distribution points can be found in Table S6, Province boundaries were taken from theChina standard map GS(2019)1822 without modification, Elevation data were downloaded from www.worldclim.org, and the Pan-Tibetan Highlands subregion boundary from [54]

The area under the curve (AUC) training value was 0.991 (Fig. S5), and all known distribution points fell into the predicted suitable habitat range, indicating that the model performed well and generated reliable evaluations. According to the current map of suitable habitat distribution, the habitat with only low suitability for *C. taliensis* was mainly predicted to be found in the Himalayas, the Hengduan Mountains, Yunnan, Taiwan and Hainan, with a few areas in the cities along the southern coast of China. Moderately suitable habitats were predicted mainly in Yunnan and Taiwan, with a few areas in southern Guangdong and the southern Himalayas in China. The highly suitable habitats were predicted mainly in Taiwan and Hainan (Fig. 7A). The predicted distribution of habitat with low suitability in the Himalayas and the Hengduan Mountains is likely to spread continually northwards towards the Tibetan Plateau in 2030 and 2090 under the SSP126 climate scenario, and the habitat with low suitability in the south coastal area will increase first and then decrease over this time. Moderately suitable habitats were predicted to decrease in the north of Yunnan in 2030 and increase in the Himalayan region in 2090 (Fig. 7B, 7C). Under the SSP585 climate scenario, the change in the predicted distribution of suitable habitat in 2030 compared with current estimates, with habitat of low suitability in the Himalayas and the Hengduan Mountains spreading to the north, and with no obvious changes in the other areas. In 2090, habitat with low suitability will spread further northwards into the Tibetan Plateau than in 2030, and low suitable habitat of low suitability in the central areas is predicted to almost disappear. The reduction in moderately suitable habitat will be more pronounced in Yunnan in 2090 than in 2030, while moderately suitable areas in the Himalayas and Hengduan Mountains will spread northward (Fig. 7D, 7E). There was never any significant change in the predicted distribution of highly suitable habitat.

In terms of area change (Table S7 and Fig. S6A-C), under the SSP126 climate scenario, both the area of habitat with low suitability and that of highly suitable habitat are predicted to increase in 2030. By 2090, the area of highly suitable habitat is predicted to shrink to below the current level, and the area of habitat of low suitability will shrink but still be higher than the current level. The area of moderately suitable habitat is predicted to have decreased by 2030 but then to increase again between 2030 and 2090, but to still be lower than the current level. Under the SSP585 climate scenario, the area of habitat with low suitability is predicted to have increased by 2030 and again by 2090, while the areas of moderately and highly suitable habitat are predicted to initially decrease and then to increase slightly, but these areas are not predicted to recover to the present extent.

## Discussion

### Population structure

The earliest described *Corybas* from Guizhou (GZ) was described as a new species, *C. fanjingshanensis* [37]. In this study, the inference of genetic structure from both PCA analysis and Bayesian cluster analysis suggested that *C. fangjingshanensis* was not an independent species, but instead should be synonymized with *C. taliensis* (Figs. 2 and 3). In order to determine its exact species, more detailed morphological studies and more in-depth phylogenetic studies are needed in the future.

**Fig. 8:**
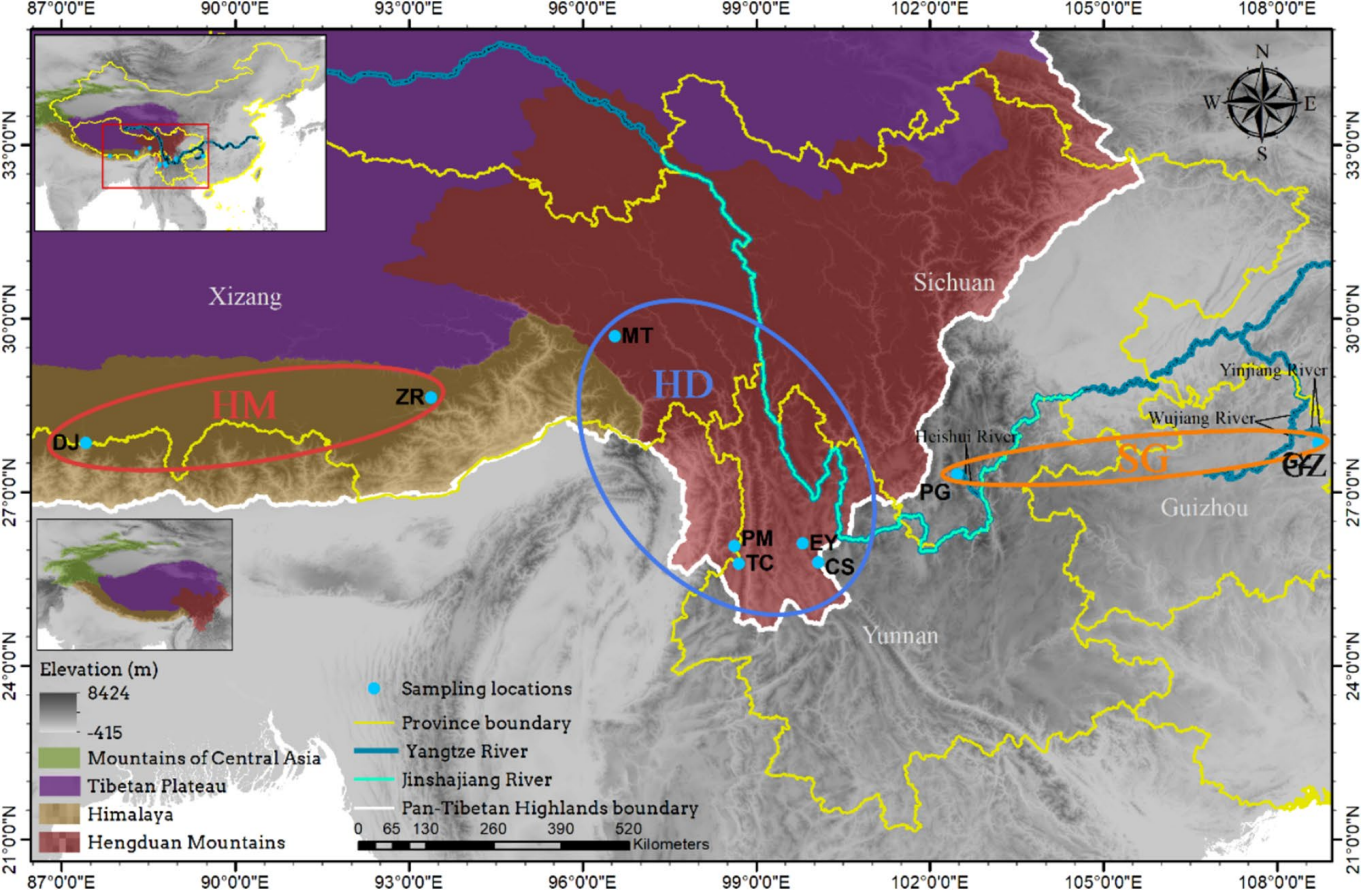
Map of all sampling locations. All of the sampling locations except for PG and GZ are in the Pan-Tibetan Highlands. ZR and DJ are in the Himalayas, and CS, EY, TC, MT and PM are all from the Hengduan Mountains. Based on PCA analysis, ZR and DJ clustered together and were defined as HM population, while PG and GZ clustered together and were defined as SG population. All the other sampling locations (CS, EY, TC, MT and PM) made up HD population. *Note*: More information about the sampling locations can be found in Table S8, Province boundaries were taken from theChina standard map GS(2019)1822 without modification, Elevation data were downloaded from www.worldclim.org, The Pan-Tibetan Highlands subregion boundary and Yangtze and Jinshajiang Rivesr were taken from [54] and [56]

We analyzed the population structure mainly based on the results of the PCA, with the Bayesian clustering analysis (*K* = 4) used as auxiliary information. Mantel tests of geographic distance, environmental distance, resistance distance and genetic distance between nine sampling locations revealed a significant pattern of IBD and IBR_env_ in *C. taliensis* (Fig. S3). There was little genetic differentiation among the sampling locations (Table 2), which is consistent with the results from PC1 and PC2 in the PCA. The observed low genetic differentiation may be because these sampling locations had the same recent origin and have not been isolated for long enough to reach high differentiation [27], or because there is some genetic communication between them [55]. The locations with the most obvious differentiation from each other were DJ and GZ, which were also the furthest apart of all the sampling locations geographically (Fig. 8), which may be one of the causes of this differentiation.

The negative Tajima’s *D* values for *C. taliensis* might indicate genes with an excess of low-frequency variation, suggesting that the populations might have experienced expansion after a recent bottleneck, the higher medians of N_1a_, N_3b_, N_1_, N_2_ and N_3_ than of N_0_ in DIYABC support the population expansion (Table 4), or that the genes targeted are under positive selective pressure [57, 58]. However, if not impossible, it is very difficult to distinguish between population growth and selection if only intraspecific polymorphism is studied. Due to the combined action of mutation, population size, recombination rate, gene conversion and selection intensity, the spectrum may be different for different genes. Comparing the intraspecific and interspecific polymorphism in homologous genes among related species is helpful to detect or confirm which genes are under selection [59–62]. Unfortunately, there are currently no data from suitable species related to *C. taliensis* available for correlation analysis. In the future, data sets from appropriate related species can be analyzed to further investigate the causes underlying the multiple negative Tajima’s *D* values.Except for the SG population, which is located in the high mountains of Sichuan (PG: Ganhaizi 2300–3200 m a.s.l.) and Guizhou (GZ: Fanjing Mountain 2300–2320 m a.s.l.), all of the other sampling locations are in the Pan-Tibetan Highlands [54, 63]. The HM population (ZR, DJ) is in the Himalayas, and the HD population (CS, EY, TC, MT and PM) is from the Hengduan Mountains (Fig. 8). The Himalaya-Hengduan Mountains (HHM) region is a significant reservoir of global biodiversity in general and for alpine plant life in particular [16, 64]. Unlike the continuous range of the Himalayas, the Hengduan Mountains constitute a series of high, parallel, mostly north–south running ridges, separated by deep valleys [65] and forming groups of sky islands. Our IBR_env_ results showed that environmental resistance may have a significant effect on the genetic differentiation of *C. taliensis*. In fact, this is a very obvious characteristic of the sky island species. Niche conservatism is hypothesized to drive diversification in sky island species and predicts that lineages should be separated by unsuitable conditions [21, 66, 67]. Sky island populations are therefore predicted to have high levels of inter-population genetic divergence and unique patterns of genetic structure [19, 66]. We found no obvious recent gene flow among the sampling locations, which might suggest that the little genetic differentiation observed in *C. taliensis* sampling locations are more likely to be as a result of ancestral polymorphism rather than to gene flow, as is the case in *Drosophila innubila* [68].

DJ and ZR of the HM population are two distant sampling locations on the same sky island. If niche conservatism drives diversification in sky island species, the observation that these two populations clustered together in a single group may result from the similar environmental characteristics of the two sampling locations on the same mountain, leading to similar levels of genetic differentiation [69].

GZ and PG of the SG population are far away from each other in spatial distance, however, they obviously clustered into one group in the PCA analysis (Fig. 3), which contradicts both the result of the Mantel test (Fig. S3) and the hypothesis of diversification in sky island species. PG and GZ in the SG population are located on different sky islands, with PG being located in the south of Sichuan and GZ located in the northeast of Guizhou. However, the Yangtze River flows through the two locations [56]. The Heishui River, a first-class tributary of Jinshajiang River (part of the upstream reaches of the Yangtze River) flows through PG, and the Yinjiang River, a tributary of the Wujiang River (a first-class tributary of the Yangtze River), flows from east to west from the south side of GZ. Recent genetic analyses suggests that intercatchment (and upstream) dispersal does occur in many plant species [70–73], so we speculate that the Yangtze River flowing through these two localities may somehow connect them, or perhaps the environmental characteristics of the two localities are similar enough that it leads to similar levels of genetic differentiation [69].

The sampling locations of the HD population (CS, EY, TC, MT and PM) were all located on different mountains of the Hengduan sky islands group, and the gene flow between these locations was complex (Fig. 2). This may be because the north–south mountain chains and valleys can provide corridors for floral exchange between the north and south but represent barriers to migration between the east and west [69, 74, 75]. In scenario 4 supported by DIYABC (Fig. 4), HM population and SG population have diverged from an ancestral population. After that, there has been an admixture event between the two populations giving birth to an admixed population (HD). Most sky island species are highly divergent between locations from different sky islands [76–78], and our population structure results therefore suggests that the *C. taliensis* populations from the Hengduan sky islands group may be much more recently established than other studied species. The current lack of geographic divergence may be due to the maintenance of ancestral variation. If this is the case, we expect that the *C. taliensis* sampling locations will become as structured as those of other species over time [68].

The HD population lies midway between the SG and HM populations, and there was evidence of small amounts of geneflow diffusion on both sides towards the SG and HM populations. It may be that these populations share a common ancestor, or perhaps there is occasionally dispersal over long distances mediated by wind or birds.

MT lies in Motuo and is relatively distant from the other sampling locations of the HD population. It is quite distinct genetically, although the Bayesian cluster analysis (K = 4) suggested that this population had a small amount gene flow from YN population (PM, TC, EY, CS). Motuo crosses many different climate and vegetation zones, and the climate of this area is complex with large vertical variation [79]. The unique geography and climate type have allowed this area to evolve a unique orchid flora [80], which may explain why MT is genetically distinct from all the other sampling locations. MT is located at the western edge of the Hengduan Mountains near the Himalayas. The PCA suggested an aggregation of the MT and YN populations (Fig. 3). This may be due to gene flow carried by the channels formed by the north-south position of the Hengduan Mountains, while the same mountains form a barrier between the MT and HM populations, blocking their east-west communication.

### Genetic diversity

Estimation of *N*_*e*_ from genetic marker data is a major focus for biodiversity conservation because it allows estimation of the rate at which inbreeding is increasing and at which additive genetic variation is lost [81]. The *N*_*e*_ values of the ZR, PM, TC, EY and GZ sampling locations were infinite (Table 3), which may have resulted from either a truly large *N*_*e*_ or as a consequence of limited data quality [82]. Low *N*_*e*_ values and low heterozygosity indicate that species have low environmental suitability, that their long-term survival is challenging and that they are at high risk of extinction [83–85]. The low *N*_*e*_ of *C. taliensis* indicated that its ability to adapt to changing environments might be low. There is a ddRAD sequencing analyses have revealed that several orchid species have very high levels of genetic diversity [86]. However, these levels of diversity, although high, were lower than those in of *C. taliensis* (Table 3) in our study. This may be due to an ancient origin of *C. taliensis* populations and/or the result of the multiple colonization of the species on different islands [87]. The *F*_*IS*_ values were significantly greater than 0, indicating that there is a possibility of inbreeding in *C. taliensis* [88]. This may be related to the field observation that *C. taliensis* may propagate asexually through tubers [44], but further experimental research is needed to confirm this phenomenon.

The differentiation among *C. taliensis* samples from different sky islands was low. This suggests one of two scenarios: the individuals in these sky islands have the same recent origin [27], or that there is some genetic communication between them [55]. BayesAss analysis showed that there was no obvious recent gene flow among sampling locations, so we believe that the former speculation is more likely. At the same time, the positive *F*_*IS*_ and field investigations by Wang show that *C. taliensis* may suffer from inbreeding, making it difficult for the genetic information of individuals in different sky islands but from the same ancestor to change obviously over a period of time, and maintaining a state of low differentiation for a long time [44, 88]. Simply put, the *C. taliensis* in each sky island may have the same recent ancestor, but to prove this conjecture, more in-depth field experiments and more detailed genetic sequencing analysis are needed in the future. It is also possible that natural selection is involved, which would lead to the evolution of similar genetic traits in similar environments, resulting in low differentiation [69]. However, we have no evidence at present that *C. taliensis* individuals from different sky islands are exposed to similar environmental conditions and face similar selection-pressure.

### Risk of non-adaptedness

In our pyRONA analysis, almost every sampling location was found to have a high RONA value, indicating that the survival of this species is vulnerable to climate change [89, 90]. The future changes to bio1 will have a negative impact on almost every sampling location, with individuals in PG, EY, GZ and MT predicted to face the most significant threats. These locations are those most likely to lose all their C. taliensis individuals in the future [91, 92].

### Predicting the potential geographical distribution

The areas of suitable habitat for *C. taliensis* are predicted to be mainly in Yunnan, the mountains of Taiwan, the Hengduan Mountains and the Himalayas in both the present and the future (Fig. 7A-E). The ranges of elevationally determined species have been shown to change with climate change [93]. On mountains around the world, increasing temperatures force alpine plants to migrate upwards until they reach the highest elevations [94]. The higher the mountain, the more future refuges for organisms under future climate change [95, 96]. The Pan-Tibetan Plateau has experienced significant warming in recent decades, which has led to large-scale glacier contraction [97, 98], lake formation and expansion [99] and extensive evolution of vegetation [100]. There are a large number of mountains with an altitude of about 4000–5000 m in the Tibetan Plateau [63], and continuous global warming will lead to further contraction of glaciers. It is likely that this will provide suitable habitat for the migration of *C. taliensis*, and this is also likely to be the reason that habitat with low suitability for *C. taliensis* in the Himalayas and Hengduan Mountain regions is predicted to spread northward into the Tibetan Plateau in the future (Fig. 7B-D, E).

Unfortunately, with climate change, species growing in high altitude areas are often caught in ‘summit traps’. This is because species inhabiting mountain summits are forced to move to higher elevations with temperature increases, and with further increases in temperature they may have no escape route and may become locally extinct [94]. The distribution of moderately suitable habitat in Yunnan is likely to contract in the future (Fig. 7E), and it may be that *C. taliensis* will be caught in a ‘summit trap’ here. In contrast, the moderately suitable habitat in the Himalayan and Hengduan Mountains regions are predicted to continue to spread, possibly due to the high altitudes of these areas offering ‘biological refuges’. The distribution of highly suitable habitat changed very little (Fig. 7A-E), which may be because it becomes increasingly difficult to find highly suitable habitat for the conservative *C. taliensis* under global climate change and human disturbance scenarios.

## Conclusions

Dispersal in *C. taliensis* is difficult, as it is a sky island species, and the future distribution of its habitat is unstable. The RONA values show that this species is vulnerable to climate change. However, the species has relatively high genetic diversity. Individuals from our nine sampling locations could be divided into three genetic groups: the SG population, the HD population and the HM population. Sampling locations of *C. taliensis* from different sky island were not highly structured, which might be related to the maintenance of ancestral variation, similar environmental characteristics of the sky islands, or transmission of seeds by wind or birds. The gene flow in the HD population was found to be more complicated than in other populations, and MT was distinct genetically from the other sampling locations due to the unique environment in Motuo. With their limited geographic range, endemic species of sky islands are particularly susceptible to climate change [101]. The results of our potential geographic distribution modeling indicated that *C. taliensis* is likely to be caught in a ‘summit trap’ in low altitude areas. However, the high altitudes of the Himalayan and Hengduan Mountains regions will act as ‘biological refuges’ for *C. taliensis.* Management and protection plans for the conservation of *C. taliensis* need to take into account the different climate changes predicted for different areas and over different periods in the future. In our field investigations, we found that some sampling locations (including PG and EY) were affected by grazing and that the *C. taliensis* plants were also sometimes collected by local people. Both of these activities can threaten the survival of plant species. At the same time, because of its low environmental adaptability and narrow habitat range, it is not feasible to do ordinary cultivation experiments on *C. taliensis*, and simple ex-situ conservation strategies are therefore currently unrealistic.

The following suggestions are therefore put forward for the protection and management of *C. taliensis.* (1) Mountain peaks behave like islands, and show high levels of genetic isolation, so we suggest that the locations of *C. taliensis* should be managed like islands, for instance promoting the use of native germplasm for regeneration efforts [32]. TC and CS are in nature reserves and are likely to be well protected, but other locations lie outside nature reserves. We suggest that part of each sky island should designated a protected area for the targeted protection and management of this species, and effective population protection monitoring should be continually carried out to prevent local extinctions caused by human disturbance and environmental fluctuations. (2) The environment and habitat preferred by *C. taliensis* should be investigated in detail, and we should aim to create a suitable environment for cultivation in an area that is convenient and reachable, so that ex-situ cultivation and conservation can be carried out. After this ex-situ conservation technology is realized, germplasm resources from PM, MT, ZR, PG, GZ and EY, all of which are greatly disturbed by humans or have only very small numbers of individuals, can be collected and grown ex situ, to ensure that the resources are preserved before the species becomes extinct at these localities [102]. PCA analysis suggested that the SG population (GZ, PG), the HM population (ZR, DJ) and the HD population (CS, EY, TC, MT and PM) represent different genetic backgrounds, and the germplasm resources from these different genetic groups need to be collected and preserved. The individuals at the YN population may face local extinction because of the ‘summit trap’, and the protection and preservation of the germplasm resources from this location is therefore a priority. The MT population represents a unique genetic group, and it is necessary to focus on the preservation of the individuals from this location, to preserve the diverse genetic resources of this species [103]. In addition, we should pay more attention to PG, EY, GZ and MT, as they are the distribution sites most likely to become extinct in the future climate change [89]. (3) The areas of potentially suitable habitat predicted in our study should be used to look for more potential distribution of *C. taliensis* in the wild, which can then be used to protect and manage further resources. (4) The populations in the Himalaya and Hengduan Mountains regions have the potential to spread further northward into the Tibetan Plateau in the future. We should investigate whether these populations have indeed spread northwards in these areas through long-term population and environmental monitoring, and should make timely investigation and protection measures [104, 105]. We believe that if the above protection and management measures are implemented, the likelihood of long-term survival of *C. taliensis* will be increased.

## Methods

### Sampling, DNA extraction and sequencing

We sampled 15 individuals at each location, witha distance between individuals of at least 10 m. A total of 135 individuals of *C. taliensis* from 9 locations (DJ, ZR, MT, PM, TC, EY, CS, PG, GZ) were sampled (Fig. 8). The environmental characteristics were recorded and the N at each sampling location was estimated while sampling. Huichun Wang conducted the plant material collection and the formal identification of *C. taliensis* for this study. One voucher specimen was collected at each sampling location and was deposited in the Germplasm Bank of Wild Species, Kunming Institute of Botany, Chinese Academy of Sciences, Kunming (code HW2020001–HW2020009, see Table S1). Permits for the collection of leaves and voucher specimens of *C. taliensis* in this study were obtained from the Motuo National Nature Reserve, the Cangshan and Erhai National Nature Reserve Management Center and the Gaoligongshan National Nature Reserve. The collection of all materials complied with the Regulations of the People’s Republic of China on the Protection of Wild Plants and the IUCN Policy Statement on Research Involving Species at Risk of Extinction.

The cetyl trimethylammonium bromide (CTAB) method was used to extract total DNA from leaf tissue [106]. The DNA integrity and concentration of each sample was assessed using agarose gel electrophoresis (Omega Bio-Tek, Norcross, Ga., United States) electrophoresis anda fluorometer (Qubit 3.0, Thermo Fisher Scientific, Waltham, Ma., United States) respectively. Each qualified DNA sample was standardized with ddH_2_O to the same volume (10 µL) and quantity of DNA (200 ng). Then, 10 µL of pre-mixed double enzyme (EcoRI and MseI; New England Biolabs, Ipswich, Ma., United States) digestion solution was added to each sample. Samples were subjected to PCR (Takara PCR Thermal Cycler Dice, Takara Bio Inc., Shiga, Japan), under the following conditions: samples were reacted at 37 °C for 8 h, followed by 20 min at 65 °C, and then finally incubated at 12 ° C. Agarose gel electrophoresis was then used to assess whether the enzyme had digested the fragments completely. The enzyme products were then ligated with EcoRI and MseI adapters containing sample specific barcodes, then fully mixed and placed in a PCR machine at 16 °C for 8 h, 65 °C for 20 min, and then 12 °C for the final incubation. An agarose gel was then used to screen barcoded samples by size (350–500 bp). Each library was then amplified to meet the concentration requirements for sequencing. Finally, paired-end sequencing was performed on an Illumina Hiseq X-Ten platform (Illumina Inc., San Diego, CA, United States), with the sequencing mode set to PE150, resulting in an average of 0.5 G data per sample.

### Processing of next generation sequencing data

The reads generated by ddRAD-seq were processed with Stacks v2.62 [53, 107]. We set process_radtags to demultiplexed and filtered the raw data, then set the len_limit to 140 bp to trim low-quality reads, and set retain_header -t to 135. Next, the ustacks, cstacks, sstacks, tsvbam, gstacks, and populations modules were run to call SNPs. We used different combinations of the parameters M (Maximum distance in nucleotides allowed between stacks), m (Minimum depth of coverage required to create a stack), and n (number of mismatches allowed between sample loci when build the catalog) for SNP calling, and finally chose the best combination of parameters by screening through the maximum number of polymorphic sites in many individuals. This process included (i) selecting the optimal m value in the range of 2–7 when default M = 2 and n = 0, (ii) selecting the optimal M value in the range of 1–5 with the m value optimized in the previous step and n = 0, and (iii) using the previously optimized M and m values, selecting the optimal n value among M − 1, M and M + 1 [108]. To reduce the rate of missing SNPs in the matrix during this process, we used the filtering parameter -r = 0.6 to ensure that no less than 60% of the individuals in the population had a particular locus. The threshold parameter -min-maf was set to 0.01 to improve the accuracy of the model-based population structure analysis [109]. To exclude closely linked loci and retain only the first SNP of each ddRAD-seq locus, we used-write-single-snp. The resulting SNP data sets were used for the subsequent analyses, during which the required data format conversion was performed using PGDSpider v2.1.1.5 [110]. Tajima’s *D* values were calculated using VCFtools v0.1.16 in 500-kb windows to test all the loci for neutrality [111].

### Population structure and genetic diversity

PCA analysis and Bayesian clustering analysis were used to deduce the population structure of *C. taliensis*. PCA analysis was performed using the R package “adegenet” [112], and confidence ellipses were drawn for each sample location including 95% of the individuals. The Admixture software was used for the Bayesian clustering analysis [113]. We tested numbers of clusters from *K* = 1 to *K* = 9 and estimated the optimal number of clusters as that with the lowest cross-validation error rate to determine the optimal value of *K*. In R v4.2.2 [114], the packages “Pophelper” [115] and “Bar Plots” function [116] were used to visualize the Bayesian clustering analysis results. Bayesian inference approach BayesAss [117] as implemented in the BA3SNP program [118] was used to assess recent gene flow between sampling locations, with 1.0 × 10^7^ iterations, a burn-in of 1.0 × 10^6^ steps, and a sampling frequency of 1000. Arlequin v3.5.2.2 [119] was used to carry out AMOVA analysis to calculate genetic differences among genetic groups, and sampling locations.

In order to test the effect of the environment on the genetic structure of *C. taliensis*, IBD, IBE and IBR tests were carried out. Pairwise geographical distances among the 9 sampling locations were computed using GENALEX v.6.5 [120], with pairwise genetic distance taken to be *F*_*ST*_/(1-*F*_*ST*_) to test for IBD. We used 19 bioclimatic variables from WorldClim v2.1 (www.worldclim.org) [121] and 44 soil factors from soil data (Harmonized World Soil Database v1.2) [122] at 30 arc-s resolution. To avoid multicollinearity, we carried out Pearson correlation analysis to eliminate one of every pair of variables with a correlation coefficient greater than 0.75 and p-value less than 0.05. Eight environmental factors were retained for analyses (Table S9). The selected environmental factors could be used to calculate the environmental distances among the 9 sampling locations. IBE can also be tested using environmental distance instead of geographic distance. Based on the circuit theory, the resistance distance was generated in CIRCUITSCAPE v.4.0.5 [123]. Here we calculated the resistance distance of the environment and the altitude respectively (environmental resistance distance and altitude resistance distance), which were then used to test IBRenv and IBRalt [124]. The R package “vegan” [125] was used to perform Mantel tests.

The π, *H*_*e*_, *H*_*o*_, *F*_*ST*_ and *F*_*IS*_ of each sampling location and genetic group were calculated using the populations module in the Stacks pipeline. The nonparametric test was carried out with the function wilcox.test in R v4.2.2 to analyze whether *F*_*IS*_ was significantly greater than 0. The linkage disequilibrium method in NeEstimator V2.1 [82] was used to estimate the *N*_*e*_ for each sample location and for each population, and a cutoff was set within the range of 1/(2n) ≤ PCRIT ≤ 1/n [126] (sample size = n).

### Demographic inference

We used DIYABC v2.1 [127] to infer population demography based on Approximate Bayesian computation. Based on the result from PCA analysis, we defined three groups: Pop1 (HM population include ZR and DJ), Pop2 (HD population include MT, CS, PM, TC and EY), Pop3 (SG population include PG and GZ). We devised four scenarios (Fig. 4), and defined t1 and t2 as the time at which differentiation may occur between groups in all scenarios, N_#_ as the effective population size in each period. The default prior values were used for all parameters. Scenario 1 is that Pop2 and Pop3 diverged from N_23_ at t1, and N_23_ and N_1_ diverged from an ancestral population (N_0_) at t2. Scenario 2 is that Pop1 and Pop3 diverged from N_13_ at t1, and N_13_ and N_2_ diverged from N_0_ at t2. Scenario 3 is that Pop1 and Pop2 diverged from N_12_ at t1, and N_12_ and N_3_ diverged from N_0_ at t2. Scenario 4 is that Pop1 and N_a_ diverged from N_1a_, Pop3 and N_b_ diverged from N_3b_, and N_a_ and N_b_ merge to produce Pop2 at the same time (t1). And N_1a_ and N_3b_ diverged from N_0_ at t2. DIYABC analysis was carried out with SNP dataset. Five thousand simulations were conducted for each scenario. We decided to use principal component analysis of the pre-evaluation scenario prior combination to check the model [128, 129].

### Risk of non-adaptedness

To infer RONA, we used the 6 bioclimatic factors screened from Pearson correlation analysis during IBE testing (Table S9). In this study, in order to account for uncertainties in the models’ assumptions, two alternative climate prediction models, a low emission scenario (SSP126) and a high emission scenario (SSP585) [130], were used to calculate a RONA value for each location in PYRONA (https://pyrona.readthedocs.io/en/latest/) [131]. In order to infer the RONA of *C. taliensis* in different periods in the future, we chose to analyze the climate data of 2030 and 2090 under the SSP126 and SSP585 scenarios.

### Data preparation for prediction of current and future distribution

The occurrence records of *C. taliensis* were obtained through the Chinese Virtual Herbarium (CVH; https://www.cvh.ac.cn/index.php), the Flora of China (www.iplant.cn/foc/), official notices, field investigation data and consultation with experts. Duplicate records were deleted. If a record had only fuzzy, county-level information, a site was chosen using the Baidu Coordinate Picking System (https://api.map.baidu.com/lbsapi/getpoint/) according to the known information, and was then optimized in LocaSpace Viewer v4 (http://www.locaspace.cn/LSV.jsp) according to the known topographic characteristics of the *C. taliensis* habitat. The distribution data were screened in ArcGIS 10.8 to ensure that the distances between the final distribution points were all more than 1 km.

Initially, we selected 55 climatic factors and 44 soil factors that may have an impact on the present and future distribution of habitat suitable for *C. taliensis*. All climate data were downloaded from WorldClim v2.1 (www.worldclim.org): 19 bioclimatic variables, 12 months of monthly total precipitation, 12 months of monthly average maximum temperature and 12 months of monthly average minimum temperature. We downloaded the relevant data for the years 1970–2000 as current climate data, and chose future climate data as predicted by scenarios that modeled either sustainable development with lower emissions (SSP126) or conventional development with higher emissions (SSP585) from the CNRM-ESM2-1 global climate change model [121]. Soil data was downloaded from Harmonized World Soil Database v1.2 [122]. All data sets had a resolution of 30 s. In ArcGis 10.8, all environmental data were clipped to China, and the number of rows and columns, cell size, extent and spatial reference of all layers were checked for consistency. In order to avoid multicollinearity of variables that can result in model over-fitting [132], we used Pearson correlation analysis to examine the cross-correlation between environmental factors, and removed one of each pair of environmental factors where the Pearson correlation coefficient was greater than 0.75 and p-value was less than 0.05.

### Predicting the potential geographical distribution

We used the MaxEnt v3.4.4 [133] to simulate potential habitat suitable for *C. taliensis* using the following parameters: random test percentage: 25%, regularization multiplier: 1, replicated run type: bootstrap, and with 10 repeats. The final prediction result gives a suitability range of 0–1for the potentially suitable habitat, and we reclassified these in ArcGis 10.8 as follows: 0–0.01 was defined as unsuitable habitat, 0.01–0.35 was habitat with low suitability, 0.35–0.65 was defined as moderately suitable habitat and 0.65–1 was defined as highly suitable habitat. The area of habitat at each level of suitability was calculated under the projected coordinate system ‘WGS_1984_UTM_Zone_47N’, and a line chart of area change was drawn.

### Electronic supplementary material

Below is the link to the electronic supplementary material.

Supplementary Material 1

Supplementary Material 2

Supplementary Material 3

Supplementary Material 4

Supplementary Material 5

Supplementary Material 6

Supplementary Material 7

Supplementary Material 8

Supplementary Material 9

Supplementary Material 10

## Data Availability

The datasets analysed during the conservation genetics study are available in the National Center for Biotechnology Information (NCBI) repository, https://www.ncbi.nlm.nih.gov/bioproject/PRJNA972527. The other datasets used during the current study are available from the corresponding author on reasonable request.

## List of abbreviations

ddRAD-seq: double digest restriction-site associated DNA-sequencing
MaxEnt: maximum-entropy model
SG population: Sichuan & Guizhou population
HD population: Hengduan Mountains population
HM population: Himalaya population
YN population: Yunnan population
DJ: Dingjie sampling location
TC: Tengchong sampling location
EY: Eryuan sampling location
ZR: Zhari sampling location
MT: Motuo sampling location
PG: Puge sampling location
GZ: Guizhou sampling location
CS: Cangshan sampling location
PM: Pianma sampling location
IBD: isolation-by-distance
IBE: isolation-by-environment
IBRenv: isolation-by-environmental resistance
IBRalt: isolation-by-altitude resisdance
bio1: annual mean temperature
bio2: mean diurnal range
bio3: isothermality
bio4: temperature seasonality
bio7: temperature annual range
bio14: precipitation of driest month
bio17: precipitation of driest quarter
RONA: Risk of non-adaptedness
CIs: confidence interval values
N: number of individuals
F_ST_: fixation index
π: nucleotide diversity
H_o_: observed heterozygosity
H_e_: expected heterozygosity
F_IS_: inbreeding coefficients
N_e_: contemporary effective population size
