# Supplementary material for: Conservation genetics and potential geographic distribution modeling of Corybas taliensis, a small ‘sky Island’ orchid species in China
Source: BMC Plant Biol. 2024 Jan 2;24:11. doi: 10.1186/s12870-023-04693-y (PMC10759615; doi:10.1186/s12870-023-04693-y)
Supplement: Supplementary file 5 — Supplementary Material 5 [file 12870_2023_4693_MOESM5_ESM.docx]

## Supplementary file

**Figures:**


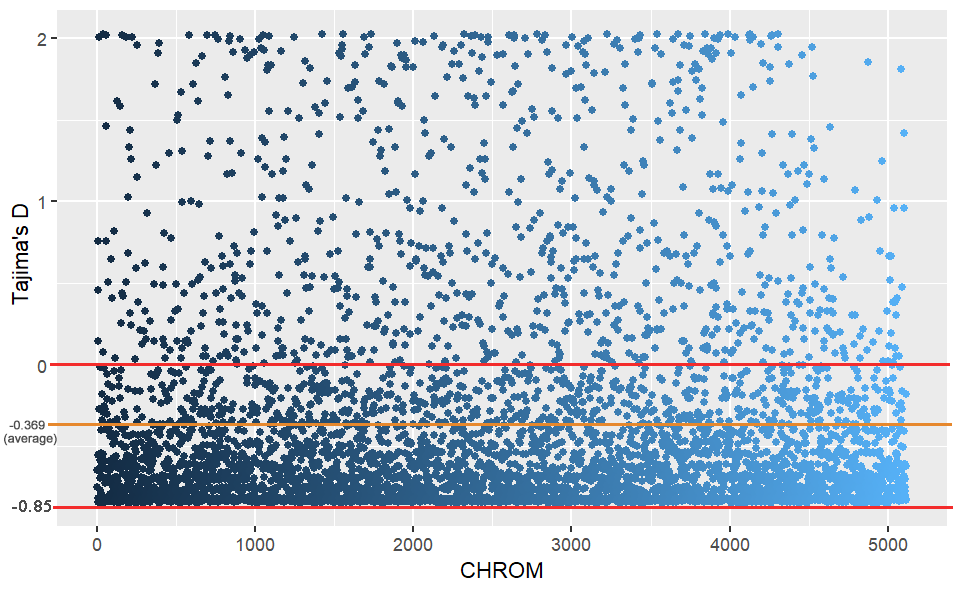
**Fig. S1**

The distribution of the Tajima’s *D* values. The maximum was 2.026, the minimum was 0.847, and the average value was 0.369. The values of 4222 among 5116 SNPs were significantly negative.


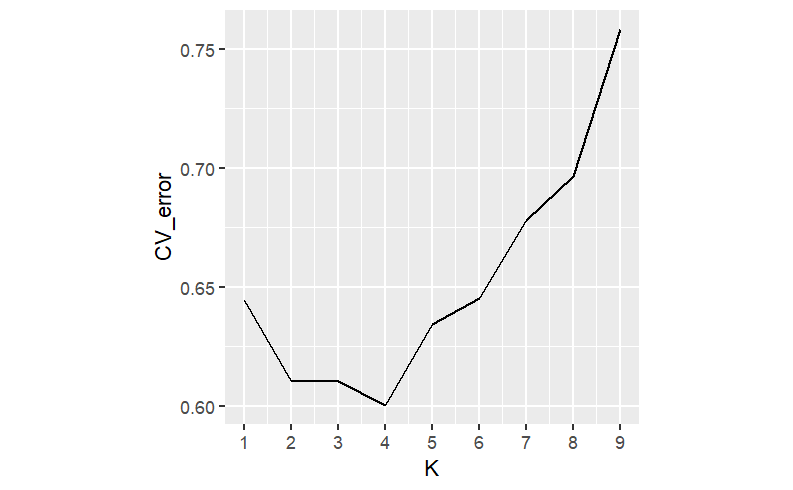
**Fig. S2**

The optimal *K* values identified using minimum CV error, the optimal *K* value was 4.

**
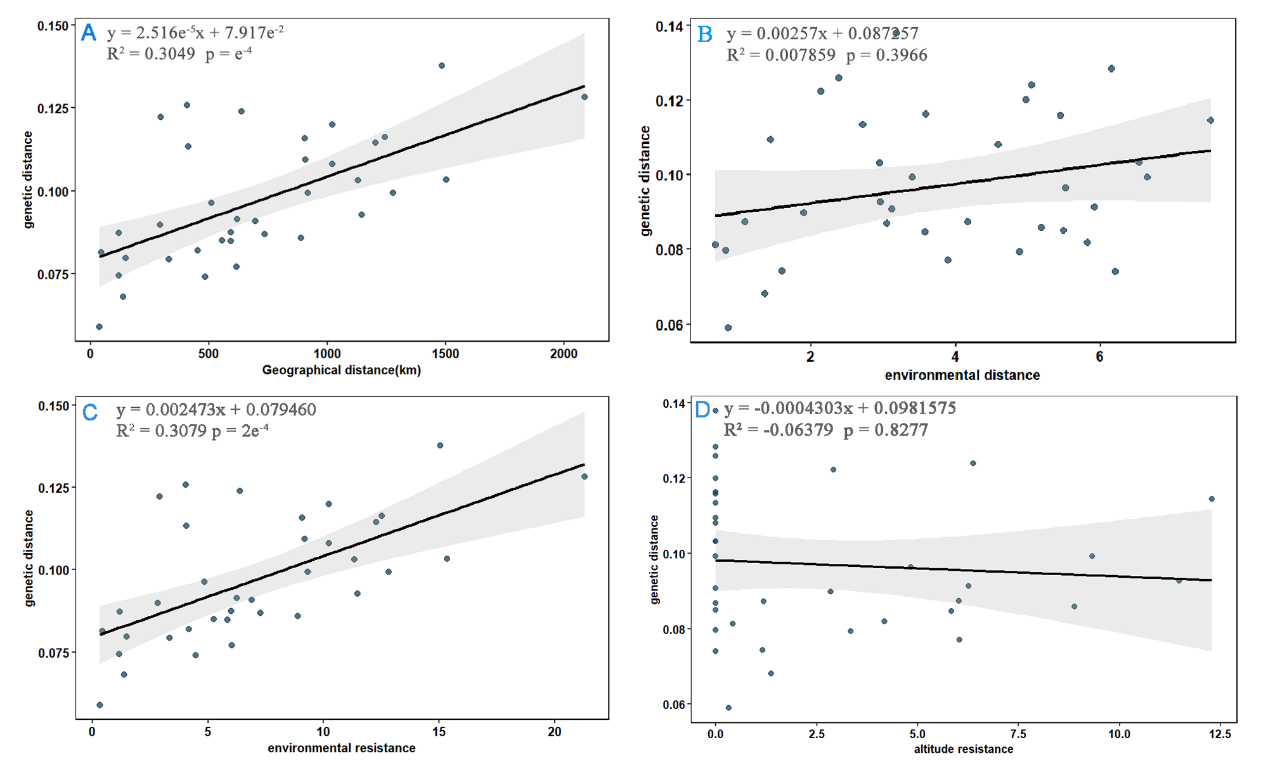
Fig. S3**

Correlations between environmental distances and genetic distance, calculated by FST/(1-FST). (A) IBD: isolation-by-distance; (B) IBE: isolation-by-environment; (C) IBRenv: isolation-by-environmental resistance; (D) IBRalt: isolation-by-altitude resistance. A and C showing the geographic distance and environmental resistance distance were significantly correlated with genetic distance.


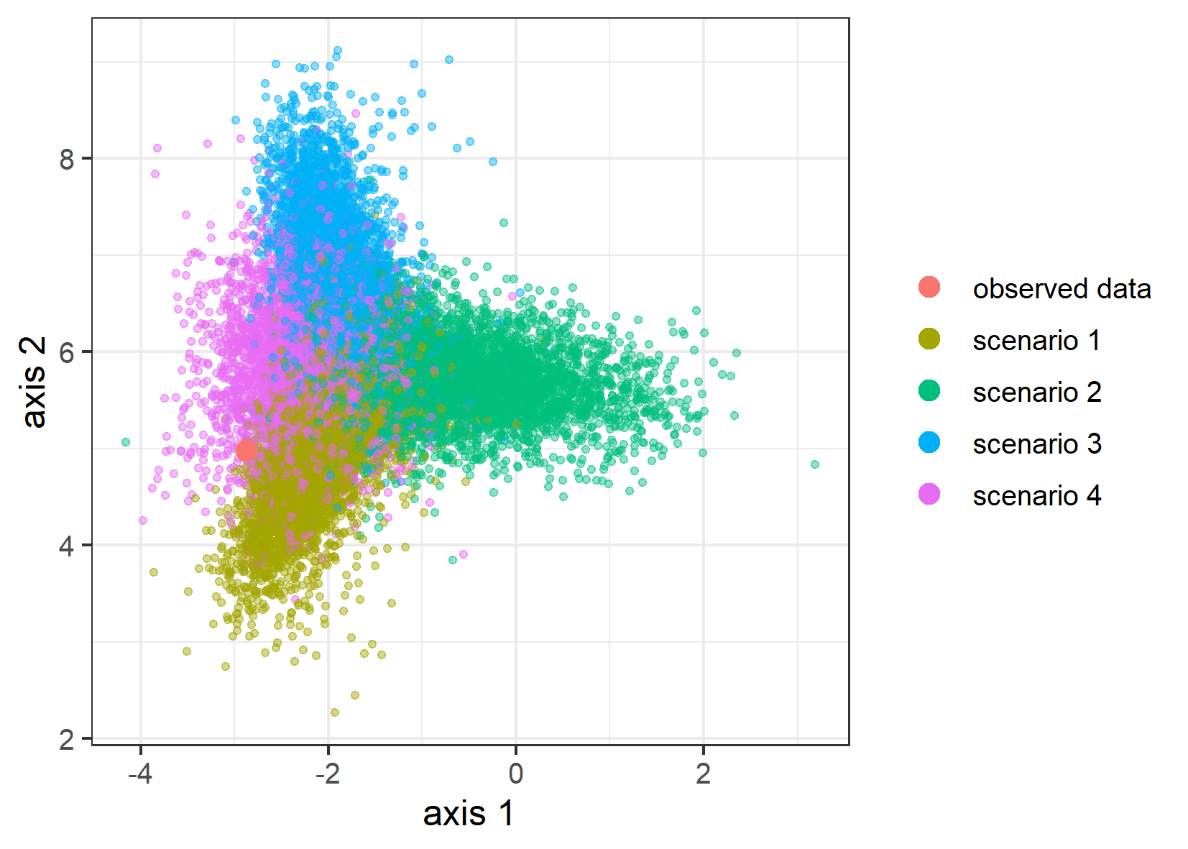
**Fig. S4**

Principal component analysis of Pre-evaluate scenario prior combination to check the model. The observed data set belongs to the point cluster of posterior distribution.

**
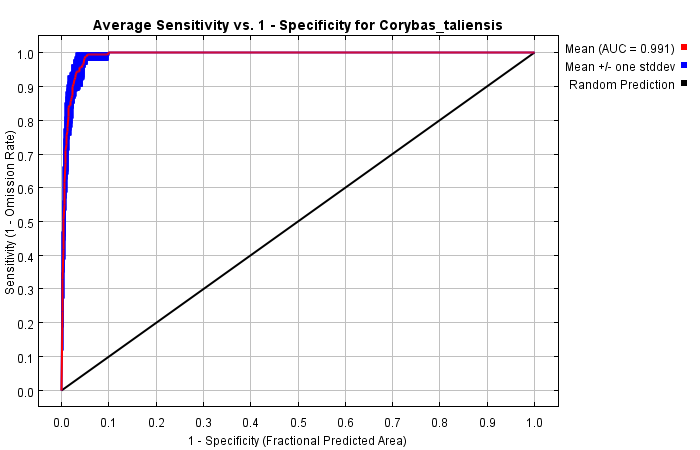
Fig. S5**

The receiver operating characteristic (ROC) curve for the same data in MaxEnt, again averaged over the replicate runs. The average training area under the curve (AUC) for the replicate runs is 0.991, and the standard deviation is 0.003.

**Fig. S6
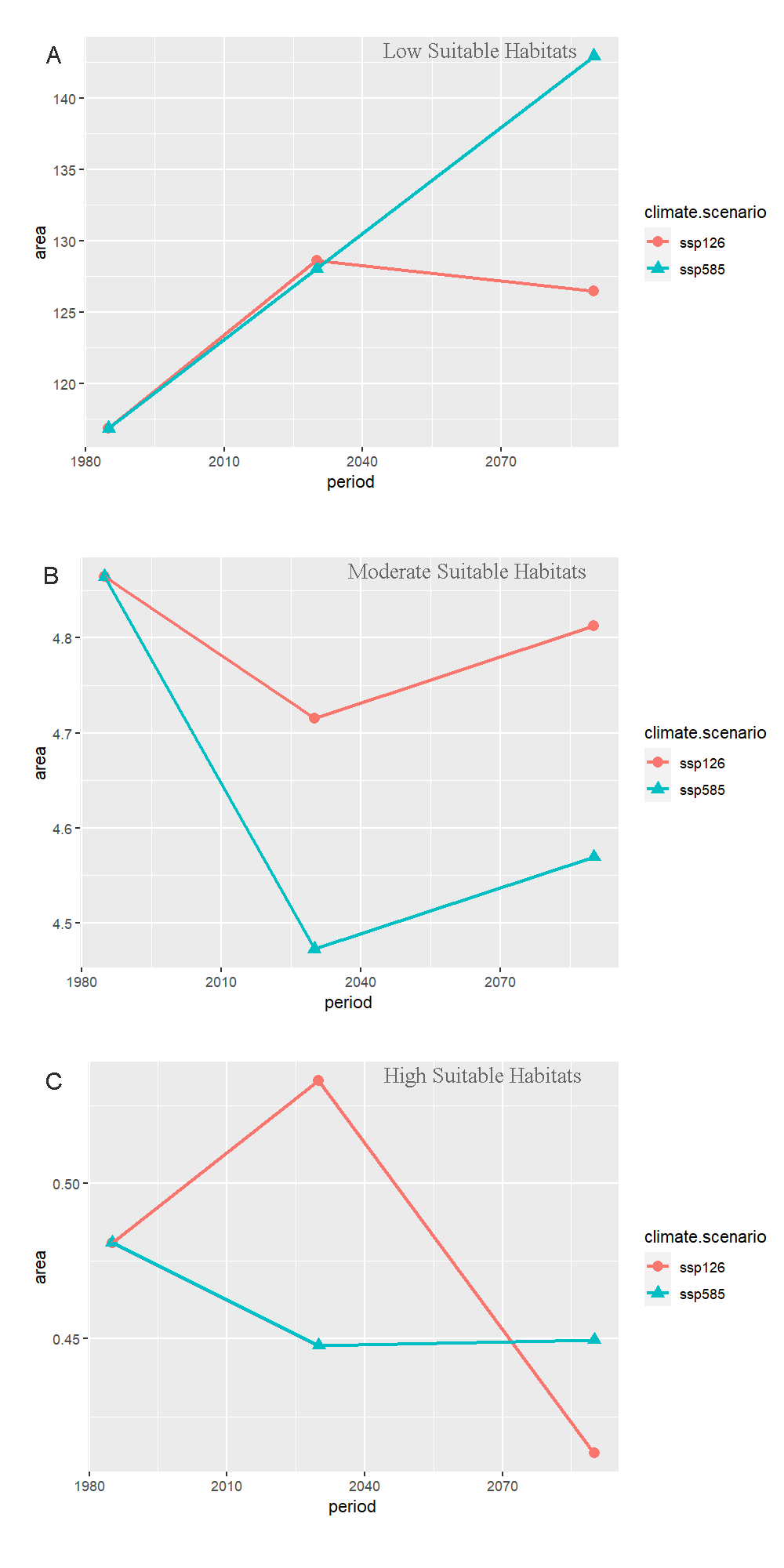
**

The change of suitable habitat area. Including low suitable habitats (A), moderate suitable habitats (B) and high suitable habitats (C) area changes in different periods under the ssp126 and ssp585 climate scenario.

**Tables:**

**Table S1** Summary of filtered and demultiplexed data of 542 million reads generated from all samples of *Corybas taliensis* in 9 sampling locations

**Table S2** The number of SNPs obtained by SNP calling under different parameter combinations, the optimal parameter combination was M = 1, m = 2, n = 2, which resulted in 5116 single nucleotide polymorphisms

**Table S3** The missing rates of individual and overall data of SNPs data that has been called out

**Table S4** The analysis of molecular variance (AMOVA) among genetic groups from PCA analysis (HM, HD and SG) and Structure by Bayesian cluster analysis (HM, MT, YN and SG)

**Table S5** Summary of Risk of non-Adaptedness of each sampling location in 2030 and 2090 under SSP126 and SSP585 scenarios, respectively

**Table S6** The 28 distribution points of *Corybas taliensis* used for modeling potential geographic distribution

**Table S7** The change of predicted suitable habitat area in 2030 and 2090 under SSP126 and SSP585 scenarios, respectively

**Table S8** The information of 9 sample distribution locations for ddRAD-seq

**Table S9** The information of 8 environmental factors selected for IBE and IBR testing
